# Exposure to permethrin or chlorpyrifos causes differential dose- and time-dependent behavioral effects at early larval stages of an endangered teleost species

**DOI:** 10.3354/esr01091

**Published:** 2021-02-11

**Authors:** Paige C. Mundy, Kara E. Huff Hartz, Corie A. Fulton, Michael J. Lydy, Susanne M. Brander, Tien-Chieh Hung, Nann A. Fangue, Richard E. Connon

**Affiliations:** 1 Anatomy, Physiology & Cell Biology, School of Veterinary Medicine, University of California, Davis, Davis, CA 95616, USA; 2 Center for Fisheries, Aquaculture and Aquatic Sciences and Department of Zoology, Southern Illinois University, Carbondale, IL 62901, USA; 3 Department of Fisheries and Wildlife, Coastal Oregon Marine Experiment Station, Oregon State University, Corvallis, OR 97331, USA; 4 Fish Conservation and Culture Laboratory, Department of Biological and Agricultural Engineering, University of California, Davis, Davis, CA 95616, USA; 5 Department of Wildlife, Fish & Conservation Biology, University of California, Davis, Davis, CA 95616, USA

**Keywords:** Delta smelt, *Hypomesus transpacificus*, Behavioral toxicology, Pyrethroid, Organophosphate

## Abstract

Pyrethroid and organophosphate pesticides are two of the most commonly used classes of insecticide worldwide. At sublethal concentrations, permethrin (a pyrethroid) and chlorpyrifos (an organophosphate) impact behavior in model fish species. We investigated behavioral effects of environmentally relevant concentrations of permethrin or chlorpyrifos on early larval delta smelt *Hypomesus transpacificus*, a Critically Endangered teleost species endemic to the San Francisco Bay Delta, California, USA. Using a photomotor behavioral assay of oscillating light and dark periods, we measured distance moved, turn angle, meander, angular velocity, rotations, thigmotaxis (time spent in the border versus center), and swim speed duration and frequency. The lowest concentrations of permethrin used in the tests (0.05 and 0.5 μg l^−1^) caused significant increases in distance moved at 72 and 96 h, respectively. At 48, 72, and 96 h of exposure, 5 μg l^−1^ of permethrin caused a hyperactive state in which the larvae significantly decreased thigmotaxis, quickly turning in short bouts of activity, characterized by significant increases in rotations and freezing events. Larvae exposed to 0.05 μg l^−1^ chlorpyrifos significantly increased thigmotaxis at 72 and 96 h. In response to 5 μg l^−1^ chlorpyrifos, larvae significantly increased velocity at 72 h exposure, and significantly increased freezing events at 96 h. Behavioral data on larval delta smelt exposed to contaminants present in their limited habitat have the potential to aid evaluations of the suitability of spawning and rearing habitats for this endangered species, thus improving conservation management strategies focused on this sensitive life stage.

## 1. INTRODUCTION

Two of the most common insecticide groups used worldwide are pyrethroids and organophosphates. Application and use of pyrethroid pesticides have grown in popularity in agricultural and urban sectors (Kuivila et al. 2012, Weston & Lydy 2012, Deanovic et al. 2018, Tang et al. 2018). Although domestic use of organophosphates has decreased due to a household ban in the USA in 2000, they are still heavily used in agriculture (Mackay et al. 2014, DiBartolomeis et al. 2019). Both categories of insecticide are of concern to aquatic organisms, and have been shown to be particularly toxic to fish (Giesy et al. 1999, Weston et al. 2015b).

Permethrin is among the most commonly detected pyrethroids in environmental and organismal samples (Tang et al. 2018). Because permethrin is used heavily in agricultural and urban applications, it often enters watersheds and can be found in sediment and surface waters in the ng l^−1^ to μg l^−1^ range (You & Lydy 2006, Delgado-Moreno et al. 2011, Weston et al. 2014, Deanovic et al. 2018). Pyrethroids are classified as Type I or Type II according to their chemical structure. Permethrin is considered a Type I pyrethroid insecticide, as it lacks an α-cyano group on the phenoxybenzyl moiety (Soderlund 2012). The mechanism of action (MOA) of permethrin is generally accepted to be the binding of voltage gated sodium ion channels (VGSCs), delaying closure of the channel, resulting in prolonged depolarization of neurons, ultimately resulting in convulsions and death (Soderlund 2012). At the whole-organism level, exposure to permethrin elicits the neurotoxic effect of tremors in mammals (Soderlund 2012, Hughes et al. 2016, Gammon et al. 2019). Pyrethroid metabolism may be species-specific (Glickman & Lech 1981), possibly contributing to the differences in tolerance between mammals and fish. At sublethal concentrations, permethrin is found to induce hyperactivity in larval fish; for example, visible spasms have been observed in zebrafish larvae at 50 μg l^−1^ (DeMicco et al. 2010). Larval zebrafish *Danio rerio* exposed to 25 μg l^−1^ permethrin decreased thigmotaxis, defined here as the affinity of an organism to preferentially remain near the boundaries of an environment, as opposed to the center (Nunes et al. 2020). Thigmotaxis has been observed in other fish species (Millot et al. 2009, Sharma et al. 2009), and can be used as a measure of anxiety in larval fish. Exposure of larval zebrafish to anxiolytic or anxiogenic compounds has been found to enhance or attenuate thigmotaxis, respectively (Schnörr et al. 2012).

Chlorpyrifos is an organophosphate pesticide, commonly used worldwide in agricultural settings (Mackay et al. 2014, Solomon et al. 2014). Because it is used heavily in the agricultural sector, it often enters watersheds, particularly after storm events, and can be found in sediment as well as surface waters (Delgado-Moreno et al. 2011, Williams et al. 2014, Weston et al. 2015a, Deanovic et al. 2018). The MOA of chlorpyrifos (in both mammals and fish) is inhibition of acetylcholinesterase, leading to accumulation of acetylcholine in the synaptic junction of neurons, ultimately causing repeated stimulation of neurons and nervous system malfunction (Giesy et al. 1999, Eaton et al. 2008). At the whole-organism level, this can lead to uncontrollable muscle twitches or spasms, eventually resulting in muscle weakness, or respiratory muscle dysfunction and/or paralysis, ultimately causing death (Jokanović & Kosanović 2010). Developmental exposure to sublethal concentrations of chlorpyrifos can cause hyperactivity and impaired choice accuracy in rats (Levin et al. 2002). Behavioral impairment observed in fish due to sublethal chlorpyrifos exposure includes decreases in total distance moved during light–dark stimuli in zebrafish (Jin et al. 2015), impaired mobility in fathead minnow *Pimephales promelas* larvae (Belden & Lydy 2006), as well as decreased swimming speed in zebrafish larvae (Richendrfer & Creton 2015) and adult medaka *Oryzias latipes* (Sastre et al. 2018). Zebrafish larvae exposed to chlorpyrifos have also been found to decrease thigmotaxis (Richendrfer et al. 2012, Richendrfer & Creton 2015).

During fish development, early larval stages are especially vulnerable to perturbations by abiotic insults, including pesticide exposures (Ramos et al. 2012). Determining the sensitivity of fish larvae to sublethal pesticide exposures is essential when assessing risks, particularly in environmental management, as these could lead to potential impacts at the population level. The delta smelt *Hypomesus transpacificus* is a euryhaline teleost species that is endemic to the San Francisco Bay Delta (SFBD). Listed as endangered species under both Federal and California State Endangered Species Acts (listed as threatened in 1993; USFWS 1993, and as endangered in 2010; CDFW 2018), delta smelt abundance has rapidly declined since the mid-1980s, and is at risk of extinction (Hobbs et al. 2017).

Delta smelt are often acclaimed as an indicator of the environmental health of the SFBD; therefore, swift drops in population abundance suggest that the ecosystem is transforming inordinately (Lessard et al. 2018). Climate change and anthropogenic manipulation of water flows are known to be drivers of change in the SFBD (Cayan et al. 2008, Cloern & Jassby 2012). Physical habitat factors, such as these, correlate strongly with the limits of habitat range in delta smelt (Brown et al. 2016). However, information regarding the impact of contaminants on early life stages of delta smelt is limited. Both permethrin and chlorpyrifos have been measured in areas inhabited by delta smelt (Weston et al. 2014, 2015a), and both have been measured at concentrations as high as the μg l^−1^ range in the SFBD, including in agricultural drainage areas adjacent to habitats in which delta smelt have been sampled (Zhang et al. 2012, Deng 2017).

One anticipated conservation goal for the delta smelt is the re-introduction of the captive refuge population into the wild (Hobbs et al. 2017, Lessard et al. 2018). The application of toxicology tests, which can be conducted under controlled laboratory conditions, will serve to inform suitable locations for release, if embryos are to be considered as a suitable life stage for re-introduction. With the UC Davis Fish Conservation and Culture Laboratory (FCCL) functioning as a conservation hatchery for some listed fish species, including delta smelt, conducting robust laboratory tests on their larvae is an opportunity not afforded to most endangered teleost species due to lack of accessibility.

Behavioral tests can be powerful tools to assess the sublethal effects of chemical compounds. Particularly, high-throughput teleost larval behavior tests can allow a quick yet thorough characterization of effects resulting from exposure to numerous classes of compounds (Miller et al. 2018, Dach et al. 2019). We adapted a photomotor test typically used to evaluate zebrafish behavior (Miller et al. 2018, Dach et al. 2019) to specifically be applied to delta smelt larvae (Mundy et al. 2020). The test involves an oscillating cycle of dark and light, which we refer to as the light–dark (LD) cycle assay. In previous studies, we have confirmed that delta smelt larvae consistently move more in the light and less in the dark, and this pattern can be perturbed by the introduction of neurotoxic pesticides (Mundy et al. 2020). Here, we used the LD cycle assay to measure differences in behavior of delta smelt larvae by analyzing multiple endpoints including distance moved, turn angle, meander, angular velocity, rotations, thigmotaxis, velocity, and swim speed duration and frequency.

## 2. MATERIALS AND METHODS

### 2.1. Fish source

Procurement and maintenance of delta smelt larvae was completed using methods described in Mundy et al. (2020). In brief, for each pesticide exposure experiment, delta smelt embryos were fertilized via strip spawning of 2 females and 2 males at the FCCL under the University of California Institutional Animal Care and Use Committee (IACUC) protocol no. 19841. Embryos were maintained at the FCCL according to their normal care conditions (freshwater and 16°C) until 7 days post fertilization (dpf) (Lindberg et al. 2013). At 7 dpf, embryos were transported to the exposure lab at UC Davis campus. Research conducted on early larval stages was approved by IACUC protocol no. 20705.

### 2.2. Chemical information

Larvae were exposed to nominal concentrations of 0.05, 0.5, and 5 μg l^−1^ of permethrin (99.5% purity, ChemService, CAS: 52645-53-1, product no. N-12848–250MG) or chlorpyrifos (99.4% purity, ChemService, West Chester, PA, USA. CAS: 2921-88-2, product no. N-11459–250MG). The concentration ranges were chosen to reflect environmentally relevant concentrations found in the SFBD and its tributaries (California, USA). Methanol (Fisher Scientific), not exceeding 0.02% v/v, was used as a vehicle solvent carrier for permethrin, while acetone (Fisher Scientific), not exceeding 0.02% v/v, was used as a vehicle solvent carrier for chlorpyrifos.

Permethrin and chlorpyrifos concentrations were measured in exposure water to confirm the nominal concentrations at the beginning of each test. The pesticides were extracted from exposure water by reversed-phase solid-phase extraction (Wang et al. 2009), and quantified using an Agilent chromatograph with a 5975-mass selective detector (MSD; Agilent Technologies) and negative chemical ionization (NCI). A complete description of the method can be found in the Supplement (Methods, Table S1) at www.int-res.com/articles/suppl/n044p089_supp.pdf. The initial concentrations were in agreement with the nominal concentration (<23% relative difference; Table S2 in the Supplement); therefore, the nominal concentrations are referenced for the remainder of the study.

### 2.3. Larval fish exposure

Compared to later life stages, larval fish are at a higher risk from exposure to lipophilic compounds, such as permethrin and chlorpyrifos, due to increased adsorption and uptake from the yolk sac. This early life stage also occurs during times of heavy rainfall, which flushes contaminants into their spawning habitat (Weston et al. 2019). For all of the tests, water quality was measured once on Day 1 (8 dpf) and once on the last day of the test (12 dpf). Temperature ranged from 16 to 16.4°C (Hanna Instruments), dissolved oxygen ranged from 9.82 to 10.43 mg l^−1^ (YSI), pH ranged from 8.55 to 8.61 (Hanna Instruments), salinity was 0.4 PSU (Hanna Instruments), and ammonia was 0 mg l^−1^ (API). Fifty percent of exposure water was renewed daily.

Exposures were conducted using methods described in Mundy et al. (2020). In brief, embryos were randomly distributed into 200 ml beakers filled with 100 ml filtered (0.22 μm) ground water with a stocking density of 20 per beaker. Embryos were placed in a temperature- and light-controlled chamber where they remained undisturbed until 8 dpf, and the start of the exposure test.

There were 4 exposure treatments — vehicle control and 3 pesticide concentrations — and 3 exposure durations (i.e. 48, 72, and 96 h). Each treatment (concentration and time) was replicated 6 times. The beakers were covered in parafilm (with several holes) to minimize evaporation, and larvae were left to acclimate in the beakers in a chamber held at 16°C and 24 h darkness.

Exposures began at 8 dpf, approximately 24 h prior to hatch so that organisms hatched into the respective treatments. Throughout the exposure, 50% of the medium was changed daily (renewed with appropriate treatment condition), at which time hatching and any mortality were also recorded. At 48, 72, and 96 h of exposure (corresponding to 10, 11, and 12 dpf), 3 larvae were removed from each replicate to perform behavioral testing (n = 15–18 larvae per treatment) (see section 2.3).

### 2.4. Behavioral assessment of pesticide-exposed larvae

In a previous study, we developed the LD cycle behavioral test that leveraged the observed natural photo motor response of the larvae (Mundy et al. 2020). At the age of interest (10, 11, and 12 dpf), 3 larvae from each replicate of each exposure treatment were carefully placed into 3 individual wells of a (non-treated) 12-well cell culture plate (Thermo Fisher no. 150200) containing 2 ml of water at specific target pesticide concentrations. Each (n = 15–18 larvae per treatment) plate was randomized such that it contained 3 larvae from each respective pesticide treatment. The larvae were allowed to adjust to the plate conditions for at least 1 h before placing the plate into a Danio-Vision Observation Chamber. Once placed in the chamber, larvae were allowed to habituate in the dark for at least 5 min. The temperature of the plate was kept at 16°C throughout the duration of the test via a recirculating water system attached to a chiller (TECO-US). Larvae were filmed from above, illuminated with programmable light from beneath the plate, set at 10 000 lux for each light cycle and infrared (IR) light during dark periods. Tracking began with 10 min dark period (Dark 1), followed by 5 min light (Light 1), 10 min dark (Dark 2), 5 min light (Light 2), and a final 10 min dark period (Dark 3). All behavioral tests were conducted between 09:00 and 18:00 h.

### 2.5. Parameters measured from the LD cycle assay

The larvae in the videos were tracked via EthoVision XT software (version 14), measuring several outputs for each larva (Table 1) including total distance moved (mm), velocity (mm s^−1^), absolute turn angle (deg), meander (deg mm^−1^), angular velocity (deg s^−1^), clockwise (CW) rotations, and counter-clockwise (CCW) rotations, all binned by minute.

The measured velocities were binned by speed into several categories including cruising (≥5 mm s^−1^ and ≤20 mm s^−1^), bursting (≥20 mm s^−1^), and freezing (≤5 mm s^−1^) using the EthoVision XT software. These speed categories were chosen to reflect categories measured in previous studies using zebrafish and fathead minnows (Steele et al. 2018), as well as preliminary studies using Delta smelt larvae (P.C. Mundy unpubl.). The categories were measured by duration (second per each minute at that speed) as well as frequency (number of times the larvae reached that speed per minute).

To measure thigmotaxis using the EthoVision software, each well was assigned an arena as the whole well (23 mm in diameter), where a smaller circle (the same size for each well) was assigned as the ‘center’ region of the well (3.5 mm from the edge of well). The area outside the center region was termed the ‘border’ region. Time spent in each region was recorded (s min^−1^). The portion of time spent in the border area or center area was calculated in R (version 3.5.3) (R Core Team 2019) by dividing seconds recorded of larvae in the border or center area by total seconds recorded of larvae in the arena (binned by minute). All data were exported from EthoVision as Excel (xls) files, and processed in R for statistics and graphing.

### 2.6. Statistics

**Fig. 1.**
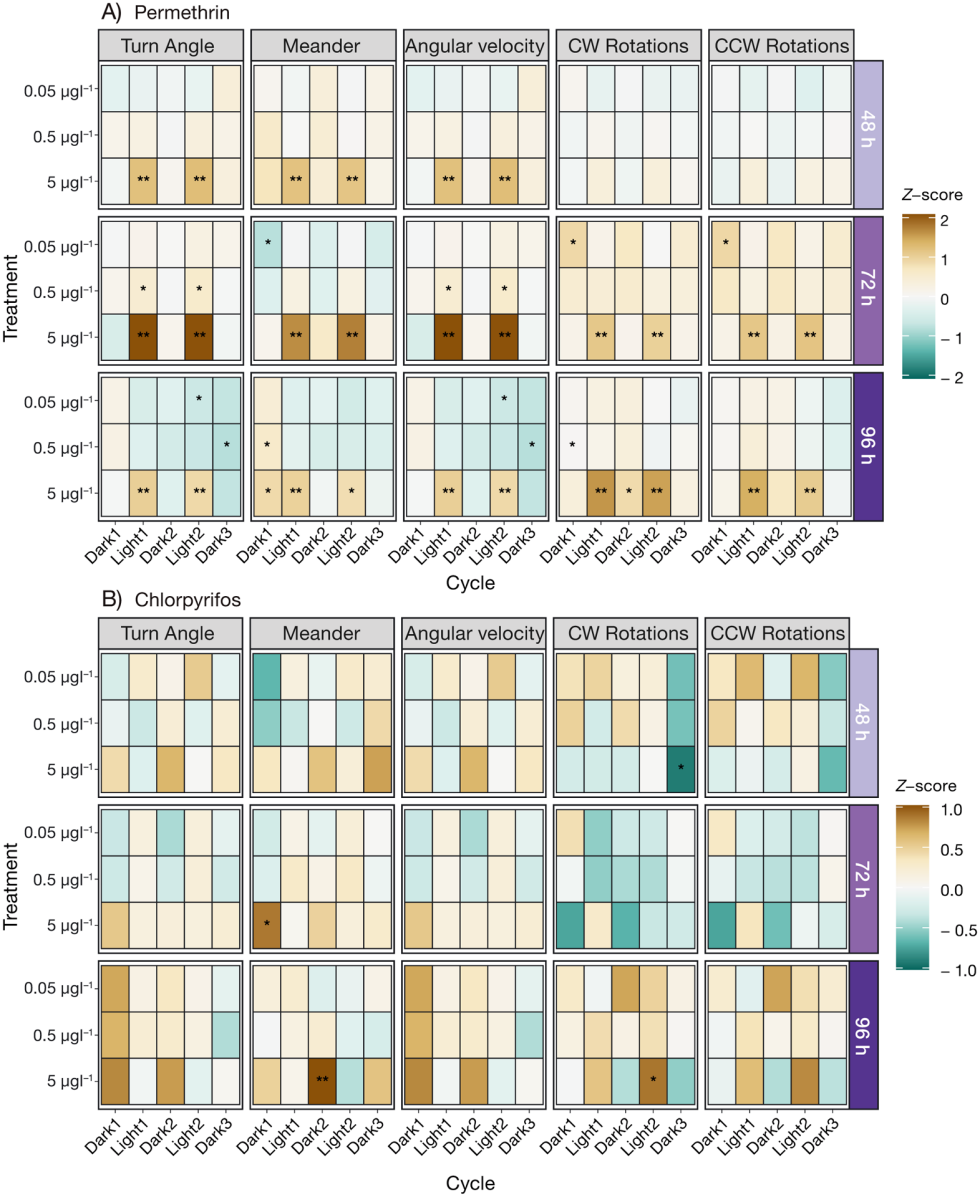
Turning of delta smelt larvae exposed to (A) permethrin or (B) chlorpyrifos. Turn angle (deg), meander (deg mm^−1^), angular velocity (deg s^−1^), clockwise (CW) rotations, and counter-clockwise (CCW) rotations are binned by minute and averaged over cycle. *Z*-score is presented for visual purposes, normalized to vehicle control. n = 15–18 larvae, *p < 0.05, **p < 0.01 (Dunnett’s test)

**Fig. 2.**
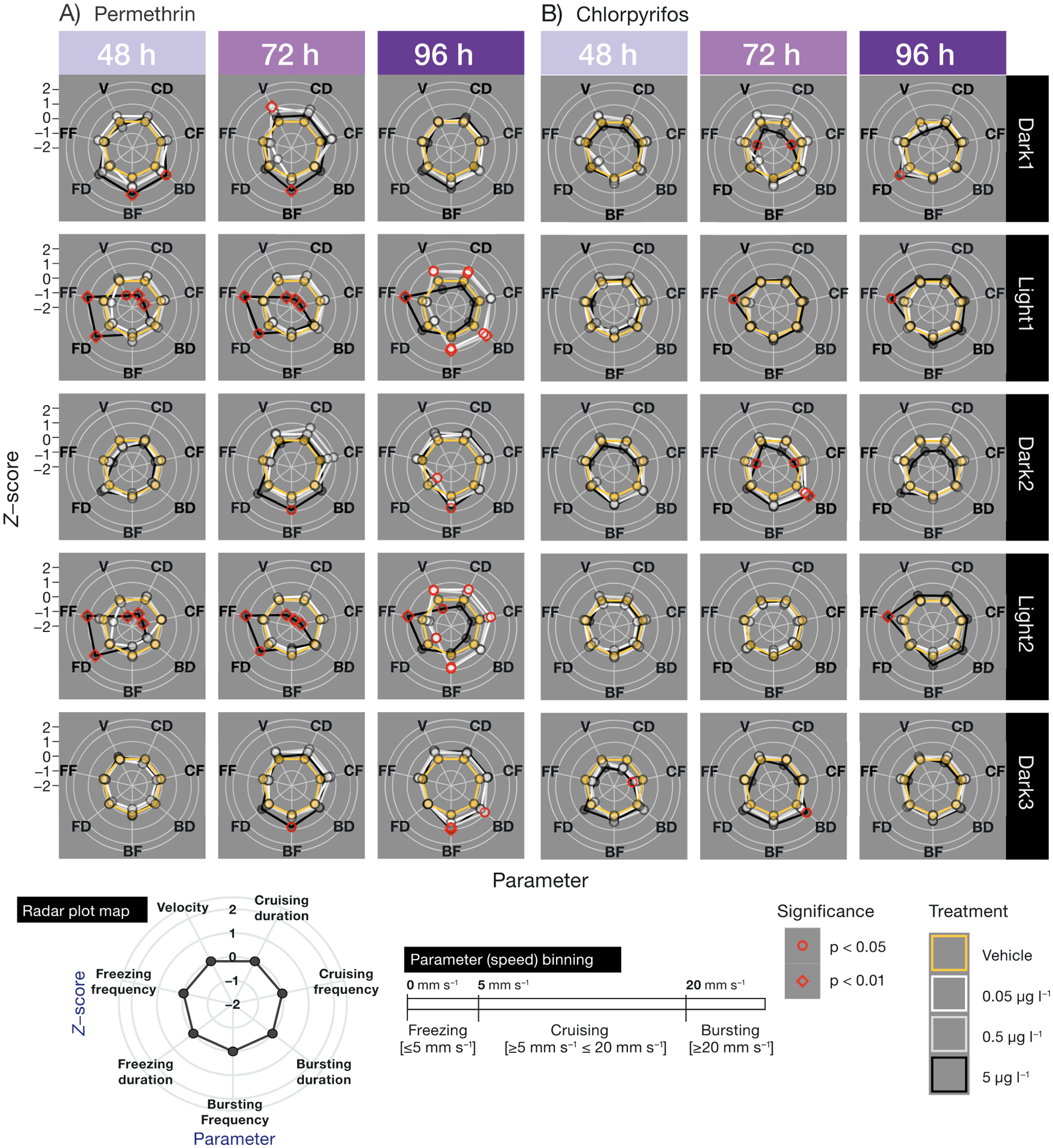
Delta smelt larvae behavioral response exposed to (A) permethrin or (B) chlorpyrifos. Response profiles are representative of each light or dark cycle. Parameters are defined as cruising (≥5mm s^−1^ ≤20 mm s^−1^), bursting (≥20mm s^−1^), and freezing (≤5 mm s^−1^). Parameters included in the graph are velocity (V; mm s^−1^), duration (time spent in the respective velocity range, s), and frequency (number of times the larvae initiated/terminated movement in a respective velocity range, count number). The plotted circles are representative of the calculated *Z*-score of each parameter (across treatments), normalized to the vehicle control. *Z*-score is presented for visual purposes, normalized to vehicle control. n = 15–18 larvae, (red circle outline) p < 0.05, (red diamond outline) p < 0.01 (Dunnett’s test)

To determine differences in parameters measured in the LD cycle assays, averages were calculated and compared within the tests per cycle. Cycle refers to the periods of dark or light including Dark 1, Light 1, Dark 2, Light 2, and Dark 3. For all behavioral analysis tests, pairwise comparisons were made only with fish from within the same batch (each batch containing a clutch from 2 females and 2 males, outlined in Section 2.1). Fish were compared only within the same dpf (exposure time), and pesticide exposure (permethrin or chlorpyrifos). Parameters were compared within each cycle (Dark 1, Light 1, etc.), and only between cycles for Fig. S1 in the Supplement. For all behavior assays, a nonparametric Kruskal-Wallis ANOVA was run to test the effects of pesticide treatment on the measured parameter using the kruskal_test function in R (Kassambara 2020). As post hoc analysis, contrasts were assessed via emmeans multiple comparison test in R (Lenth 2019), using the contrast method (control versus treatment) to compare vehicle control with the 0.05, 0.5, and 5 μg l^−1^ permethrin or chlorpyrifos treatments (α < 0.05). The p-value was adjusted using the dunnetx method (Dunnett’s test) for 3 tests (vehicle control versus the 0.05, 0.5, and 5 μg l^−1^ permethrin or chlorpyrifos treatments). To measure differences in movement between cycles for each pesticide at each exposure time (Fig. S1), the Dunn’s multiple comparisons test was utilized via the dunns function in R (Kassambara 2020). While presenting multiple parameters having different units (i.e. mm s^−1^, counts, and s; in Figs. 1 & 2), the *Z*-score is presented, normalized to vehicle control to increase visual clarity. The calculation of the *Z*-score was conducted using the following equation: *Z* = *(x* − μ) / σ, where *x* is the value, μ is the mean, and σ is the standard deviation.

To measure effects on total distance moved in a dose-responsive manner, the data were analyzed using regression analyses to fit concentration–effect curves based on an approach developed in Brander et al. (2016) and also applied in Frank et al. (2019) and Mundy et al. (2020). A maximum likelihood estimate (MLE) approach was used to evaluate whether non-monotonic curves were a better fit to the data than a null (intercept-only) model. Five different concentration–effect curves (linear regression, quadratic, sigmoidal, 5-parameter unimodal, and 6-parameter unimodal) were tested to fit responses of all 3 concentrations and vehicle control. A maximum likelihood ratio test was used to examine whether each curve provided a better fit than an intercept -only null model with a significance level of α < 0.05. All calculations for the concentration–effect curves were performed using mean total distance moved, rescaled between 0 and 1 within each cycle to facilitate comparison between pesticide concentration within each cycle. R scripts used for data preparation, statistical analysis, and graphing can be found at https://github.com/insideafish/larvae_behavior.

## 3. RESULTS

### 3.1. Effects of permethrin on larval delta smelt behavior

**Fig. 3.**
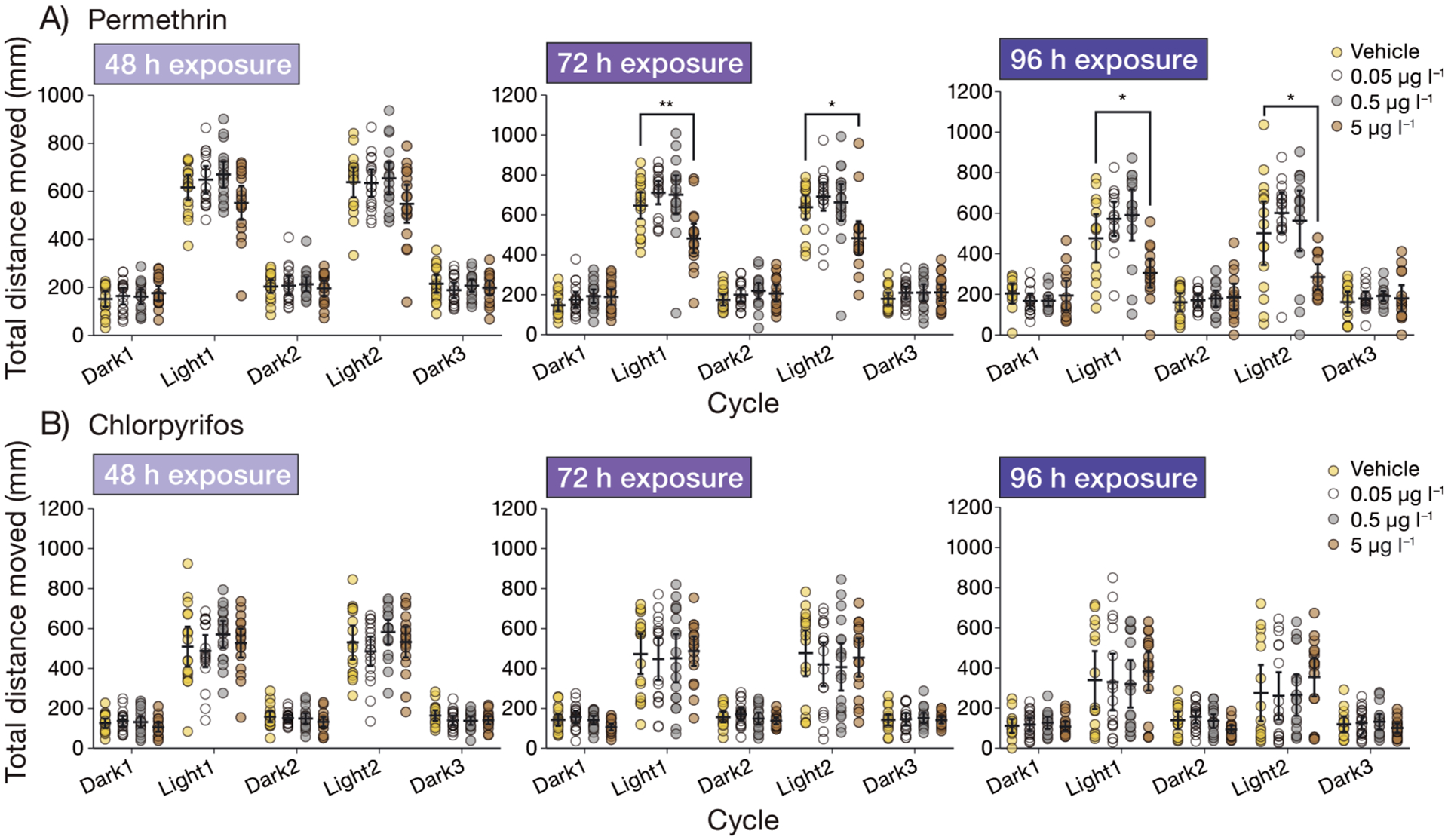
Total distance moved in the light–dark (LD) cycle assay during exposure to (A) permethrin or (B) chlorpyrifos. Mean total distance moved over each cycle, of delta smelt larvae at 48, 72, or 96 h of exposure (which correspond to 10, 11, or 12 days post fertilization [dpf]). Individual points represent larvae (n = 15–18), and bars represent mean and 95% confidence intervals. *p < 0.05, **p < 0.01 (Dunnett’s test, in comparison to vehicle control)

**Fig. 4.**
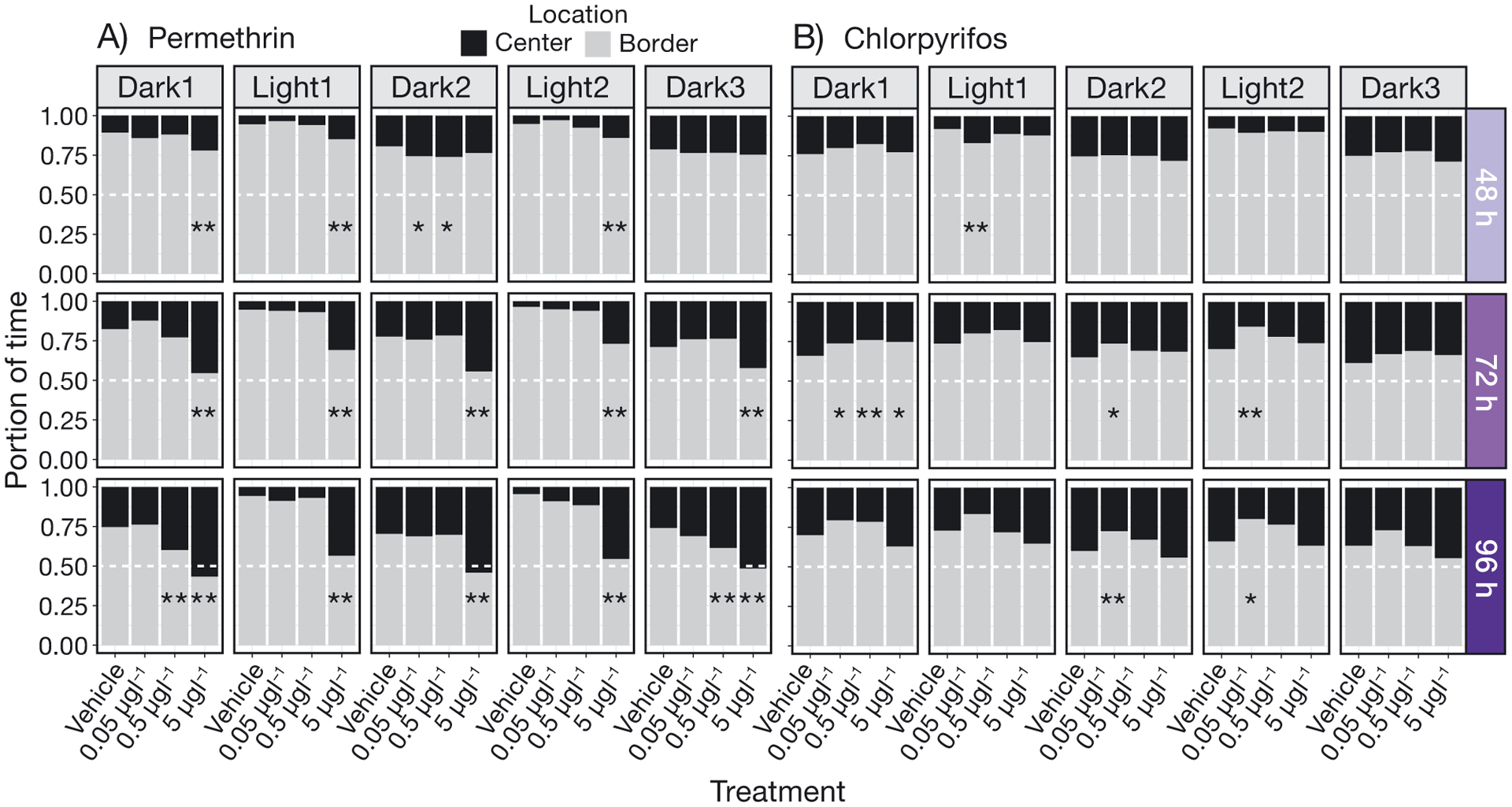
Thigmotaxis of delta smelt larvae exposed to (A) permethrin or (B) chlorpyrifos. Portion of time spent in the border or center area of the well during the LD cycle assay. Portion of time calculated as seconds recorded of larvae in the border area, and seconds recorded in the center area, divided by seconds recorded of larvae in total, binned by minute and averaged over cycle. n = 15–18 larvae, *p < 0.05, **p < 0.01 (Dunnett’s test, in comparison to vehicle control)

Permethrin concentrations as low as 0.05 μg l^−1^ significantly impacted larval delta smelt behavior (Figs. 1A & 2A), in that the larvae increased turning, as well as bursting frequencies and duration during the light periods. All statistically significant behavioral results are summarized in Table 2. In brief, at 72 and 96 h of exposure to 5 μg l^−1^, larvae moved significantly less in comparison to controls (Fig. 3A) during the light periods, and decreased thigmotaxis (Fig. 4A) as well as turning (Fig. 1A). Larvae exposed to 5 μg l^−1^ also exhibited increased bursting frequencies and freezing durations during the light periods (Fig. 2A).

When each concentration was analyzed separately for differences in movement between cycles, almost all doses exhibited a paradigm in which the larvae move more during the light periods than in the dark periods (p < 0.05, Dunn’s *t*-test). Exceptions to this paradigm were observed in 96 h of exposure to 5 μg l^−1^ permethrin, in which no significant difference in total distance moved was observed between cycles (Fig. S1).

Additionally, the data exhibited non-monotonic responses, including quadratic (Light 1 of 48, 72, and 96 h and Light 2 of 48 and 72 h) or sigmoidal (Light 2 of 96 h) dose–response relationships during the light periods of the LD cycle assay (p < 0.05) (Fig. S2).

### 3.2. Effects of chlorpyrifos on larval delta smelt behavior

Chlorpyrifos concentrations as low as 0.05 μg l^−1^ significantly impacted larval delta smelt behavior (Fig. 4B) in that they increased thigmotaxis during the dark and light periods at 72 h of exposure. All statistically significant behavioral results are summarized in Table 3. Interestingly, more differences were observed in the dark periods than in the light periods. Larvae exposed to 5 μg l^−1^ for 72 h increased bursting durations and decreased cruising frequencies during dark periods, and increased freezing frequencies during the light periods at 96 h of exposure (Fig. 2B).

Movement of larvae between cycles per concentration consistently showed increased total distance moved in the light and decreased movement in the dark periods (p < 0.05, Dunn’s *t*-test). Exceptions to this paradigm were observed in 96 h of exposure to vehicle, 0.05, or 0.5 μg l^−1^ chlorpyrifos, in which no significant difference in total distance moved was observed between cycles (Fig. S1). When analyzed for dose–response, the data exhibited decreasing linear dose–response relationships during Dark 1 at 72 h and Dark 2 at 96 h (p < 0.05) (Fig. S2).

## 4. DISCUSSION

The primary purpose of this investigation was to determine whether, and to what extent, exposure to environmentally relevant concentrations of permethrin or chlorpyrifos altered the behavior of early larval delta smelt. Several robust patterns of hyperactivity were exhibited by larvae exposed to 2 pesticides of different classes. The wide range of observed behavioral effects suggests that (1) contaminants known to be present in delta smelt habitats can alter larval behavior, and (2) the LD cycle assay can gather intricate yet comprehensive results relevant to physiological consequences of interaction with particular compounds as well as conservation efforts for delta smelt and other endangered teleost species.

The LD cycle assay is designed to be non-invasive, potentially eliminating confounding excess stress associated with handling, so as to effectively explore the minutiae of behavioral differences in these little-studied larvae. This allows the quick yet thorough characterization of effects resulting from exposure to numerous classes of compounds. Al though the parameters (e.g. light cycle lengths and light intensity) of the LD cycle assay used in the present study were specific to eliciting the most robust and repeatable outcomes in delta smelt larvae (Mundy et al. 2020), the results can be applied outside of the delta smelt in the context of elucidating and describing the variety and mechanisms of effect of the compounds.

The way in which the data were collected and analyzed in the present study, consisting of 8 collected parameters, allows for a quantification of a behavioral pattern more complex and informative than the singular parameters themselves. For example, the hyperexcitability observed in response to the highest concentration (5 μg l^−1^) of permethrin during the light cycles consisted of the larvae spending more time in the center of the well, quickly turning and rotating in bouts of increased velocity (Figs. 1A, 2A, 4A). Although overall activity may suggest hypoactivity due to decreased total distance moved within the light cycles (Fig. 3A), the quantification and assessment of other variables (thigmotaxis, turning, and velocity) (Figs. 1A, 2A, 4A) reveal the hyperactive nature of the behavior. Decreases in total distance moved during stimulatory periods in response to permethrin exposure have been observed in other fish larvae (Xu et al. 2018). In our observations, only when paired with additional results including increased turning, meander, angular velocity, rotations, and anti-thigmotaxis does this lead to conclusions of bouts of twisting, away from the border area.

In contrast with the reactions during the light periods in permethrin exposure, the most notable differences in behaviors induced by chlorpyrifos exposure occurred during the dark periods. During Dark 1 of 72 h of exposure to 5 μg l^−1^ chlorpyrifos, the larvae moved erratically at the edges of the well, quantified by increased thigmotaxis and meander (Figs. 1B, 4B). Perhaps the behavior is indicative of exploration avoidance, as increased thigmotaxis is considered an indication of anxiety-like behavior (Schnörr et al. 2012). Then, in Dark 2 and Dark 3, the periods occurring after the stimulus periods of Light 1 and Light 2, the larvae moved quickly for longer periods of time once the stimulus was removed, perhaps in a delayed reaction (Fig. 2B). The exertion of increased movement in response to the light stimulus could be over-stimulating for the exposed fish, and the stress presented as hyperactivity (bursting, etc.) in the sequential dark periods.

We observed a wide range of behavioral effects when evaluating exposure to 2 different classes of compounds at environmentally relevant concentrations, suggesting that the LD cycle assay can be a sensitive tool to gather information possibly transferable to conservation efforts. For example, future studies exposing larvae to environmentally derived, aqueous grab samples could serve to elucidate site-specific information, providing data on delta smelt-specific habitat viability. Because the delta smelt are progressively losing viable habitat due to anthropogenic factors, the ability to accurately assess habitat quality specific for this species is increasingly relevant for conservation efforts. In terms of the specific behaviors observed in the present study, behavior responses such as increased thigmotaxis, especially when observed with increased meander during chlorpyrifos exposure, could be advantageously used as test endpoints to evaluate the suitability of larval rearing habitats. If this behavior was performed for long periods in an aquaculture or environmental setting, this direction of movement could place pressure on the jaw, giving rise to craniofacial malformations, as this is a period of rapid growth. This type of outcome has been observed in aquaculture studies, including larval *Anabas testudineus* (climbing perch) housed in tanks of bright colors, that increased thigmotaxis, ultimately leading to jaw malformations (Ahmadi 2018). Outside of aquaculture, incidences of population-specific jaw malformations with unknown cause(s) have been observed in wild teleosts (Yamamoto et al. 2013).

Two of the most identifiably abnormal behaviors described in response to permethrin and chlorpyrifos (rapid twisting away from the well-edge, and erratic movement against the well-edge, respectively) occurred at environmentally relevant concentrations of pesticides. In an ecological context, the altered behavior observed in the present study could have population-level impacts. Behavioral abnormalities induced by environmental stress, including sublethal contaminant exposure, have been found to produce effects at the population and community levels in aquatic systems (Fleeger et al. 2003, Rohr & Crumrine 2005, Söffker & Tyler 2012). The indirect effects of behavioral abnormalities are often extrapolated to and studied as predator–prey interactions (Fleeger et al. 2003). For example, exposure to environmental stressors could potentially cause delta smelt living in the wild to be more susceptible to predation, or obstruct optimal prey acquisition. Experiments on juvenile delta smelt show that abnormal behavior caused by thermal stress correlates with increased predation by largemouth bass (Davis et al. 2019), a species invasive to the habitat of the delta smelt. Although no predator–prey interaction studies in the context of confounding environmental stress have been conducted on delta smelt larvae thus far, pyrethroids and organophosphates have been shown in the literature to be able to alter biological interactions in other teleost larvae. For example, increased predation risk occurred in fathead minnow larvae exposed to 0.455 and 1.142 μg l^−1^ esfenvalerate (a pyrethroid) (Floyd et al. 2008), and zebrafish larvae exposed to 2.2 μg l^−1^ chlorpyrifos were shown to be slower to habituate to a repetitive vibration stimulus (Faria et al. 2020).

Predation, without exacerbation from pesticide exposure, is already cited as a potential threat to the longevity of the delta smelt species (Hobbs et al. 2017). For example, the invasive fish species *Menidia audens* (Mississippi silverside) inhabits overlapping areas with delta smelt breeding and spawning grounds, and has been shown to prey on larval delta smelt (Schreier et al. 2016). The permethrin-induced hyperexcitability observed in the present study could have ecological impacts such as increased risk of predation due to inability to complete normal escape behavior. Because the role of larval escape response in predator–prey interactions is complex and context-dependent (Domenici 2010), more targeted studies would be necessary to fully assess the ecological impacts of the observed behavior in terms of predation. The high-throughput nature of the LD cycle as say can provide direction on which exposure concentrations to explore in more targeted studies.

Similarly, changes in abundance and distribution of prey (zooplankton) in the SFBD are predicted to play a role delta smelt declines (MacNally et al. 2010). It is generally accepted that the larval stage exists as one of the main bottlenecks predictive of the success of year classes within a fish stock, correlated with the ability to successfully obtain food in the critical time directly after yolk absorption (Leggett & Deblois 1994, Houde 2008). Therefore, navigating prey capture confounded by pesticide-induced behavioral defects could potentially escalate the consequences of diminished prey availability.

Although delta smelt are currently difficult to find in the wild (Hobbs et al. 2017), having access to a cultured refuge population allows us to develop robust laboratory tests. This situation offers a unique opportunity to gather large amounts of information on a critically endangered species. The unique situation in which delta smelt larvae are able to be cultured and studied in the laboratory is one not afforded to all endangered teleost species. Considering the compounds evaluated are used worldwide, using tests developed on delta smelt larvae can possibly give rise to information applicable to other endangered teleost species which may encounter these compounds. Including behavioral endpoints in predictive models could strengthen risk assessments when evaluating the ecological impact of contaminants (Peterson et al. 2017).

Behavioral responses to permethrin were dose-dependent (Fig. S2) in the present study, in a quadratic or sigmoidal fashion during the light cycles at all time points. Non-monotonic dose–response relationships have been observed in older (~43 d post hatch) delta smelt exposed to permethrin (Jeffries et al. 2015), in that the gene responses of smelt exposed to concentrations lower than the lowest observed effect concentration (LOEC; reported at 2.56 μg l^−1^) differed in directional changes from those at higher concentrations. Non-monotonic responses may occur for a variety of reasons, including mechanisms involving endocrine disruption. Considering that the metabolites of permethrin are more potent in terms of estrogenicity than the parent compound (Nillos et al. 2010), it could be possible that the ability to metabolize permethrin is increased at lower concentrations. The observation of non-monotonic dose–response patterns in permethrin-induced behavior across the life stages of this non-model species can be informative to those designing experiments using other endangered or sensitive teleost species in terms of selection of dosing paradigms.

Although the response to the LD cycle assay is robust and consistent as currently studied, more investigation on the life history and general physiology of delta smelt larvae is needed in order to develop best practices for toxicological screenings. We found differences in responses between the control fish for the tested pesticides and postulate that this is likely influenced by seasonality. Two separate batches were used for each test, with permethrin tested on organisms from mid-season spawns, and chlorpyrifos conducted toward the end of the breeding season (Moyle et al. 2016). Differences in a spawn’s reaction to environmental stressors across breeding seasons has been reported for other fish species (e.g. Atlantic cod *Gadus morhua*; Oomen & Hutchings 2015).

The range of responses observed in the present study supports that the behavioral effects of permethrin or chlorpyrifos are dose-dependent, as well as time-dependent, and that they occur at environmentally realistic exposure concentrations. This also demonstrates the need to carefully consider the measured variable when evaluating the effects of contaminants on behavior. Contemplating all of the variables measured together in a holistic manner may help create a picture of behavior more relevant to physiological outcomes, as well as serve as an initial litmus test for compounds to investigate more thoroughly.

## 5. CONCLUSIONS

Measuring a wide range of variables, and evaluating when they change throughout the LD cycle assay, allows a thorough documentation of behavioral effects due to sublethal exposure to contaminants. Because delta smelt larvae perform consistently in the LD cycle assay, and it is possible to glean minute behavioral differences within the test, the test can be applied to any number of compounds, allowing a ‘behavioral library’ to be built using consistent protocols and measurements. Considering that one of the goals in the conservation of the delta smelt is to reintroduce this endangered species into the wild, the results from this study and future studies can serve to inform conservation managers on the timing and location of potential effects relative to seasonality of contaminant application and presence in the SFBD. Although delta smelt play a niche role in a confined habitat, the ability to carry out in-depth behavioral studies may also give rise to information for other endangered teleost larvae that are not as easily accessible (e.g. not able to be cultured or maintained easily in the laboratory for a 96 h toxicology test).

## Supplementary Material

Supplemental Figure 1

Supplemental Figure 2

Supplementary Material

## Figures and Tables

**Table 1.** Variables measured in the light–dark (LD) cycle assay

Measurement	Unit	Calculation
Total distance moved	mm	Per minute, binned by cycle
Thigmotaxis	portion	Total seconds tracked per minute divided by seconds spent in border or center, binned by cycle.
Turn angle	Degree (°)	Per minute, binned by cycle
Meander	° mm^−1^	Per minute, binned by cycle
Angular velocity	° s^−1^	Per minute, binned by cycle
Clockwise rotations	Count	Per minute, binned by cycle
Counter-clockwise rotations	Count	Per minute, binned by cycle
Velocity	mm s^−1^	Per minute, binned by cycle
Cruising duration (≥ 5 mm s^−1^ & ≤ 20 mm s^−1^)	s	Seconds spent at that speed per minute, binned by cycle
Cruising frequency (≥ 5 mm s^−1^ & ≤ 20 mm s^−1^)	Count	Counts per minute, binned by cycle
Bursting duration (≥ 20 mm s^−1^)	s	Seconds spent at that speed per minute, binned by cycle
Bursting frequency (≥ 20 mm s^−1^)	Count	Counts per minute, binned by cycle
Freezing duration (≤ 5 mm s^−1^)	s	Seconds spent at that speed per minute, binned by cycle
Freezing frequency (≤ 5 mm s^−1^)	Count	Counts per minute, binned by cycle

**Table 2.** Permethrin behavioral results. Arrows indicate direction of change in comparison to vehicle control (↑increased or ↓decreased).

Parameter	Dark1	Light1	Dark 2	Light2	Dark 3	Exposure Time (h)	Concentration (µg l^−1^)
Thigmotaxis			↓*			48	0.05
Meander	↓*					72	0.05
Clockwise rotations	↑*					72	0.05
Counter-clockwise rotations	↑*					72	0.05
Velocity	↑**					72	0.05
Turn angle				↓*		96	0.05
Angular velocity				↓*		96	0.05
Velocity		↑*		↑*		96	0.05
Cruising duration		↑*		↑*		96	0.05
Cruising frequency				↑*		96	0.05
Bursting duration				↑*		96	0.05
Bursting frequency		↑*		↑*		96	0.05
Thigmotaxis			↓*			48	0.5
Turn angle		↑*		↑*		72	0.5
Angular velocity		↑*		↑*		72	0.5
Thigmotaxis	↓**				↓**	96	0.5
Turn angle				↓*	↓*	96	0.5
Angular velocity				↓*	↓*	96	0.5
Velocity		↑*		↑*		96	0.5
Cruising duration		↑*				96	0.5
Bursting duration		↑**			↑*	96	0.5
Bursting frequency		↑**		↑*	↑*	96	0.5
Freezing duration			↓*	↓*		96	0.5
Thigmotaxis	↓**	↓**		↓**		48	5
Turn angle		↑**		↑**		48	5
Meander		↑**		↑**		48	5
Angular velocity		↑**		↑**		48	5
Velocity		↑*		↑**		48	5
Cruising frequency		↑**		↑**		48	5
Bursting duration	↑*					48	5
Bursting frequency	↑**					48	5
Freezing duration		↑**		↑**		48	5
Freezing frequency		↑**		↑**		48	5
Distance moved		↓**		↓*		72	5
Thigmotaxis	↓**	↓**	↓**	↓**	↓*	72	5
Turn angle		↑**		↑**		72	5
Meander		↑**		↑**		72	5
Angular velocity		↑**		↑**		72	5
Clockwise rotations		↑**		↑**		72	5
Counter-Clockwise rotations		↑**		↑**		72	5
Velocity		↓**		↓**		72	5
Cruising duration		↓**		↓**		72	5
Cruising frequency		↓**		↓**		72	5
Bursting duration						72	5
Bursting frequency	↑**		↑*		↑*	72	5
Freezing duration		↑*		↑*		72	5
Freezing frequency		↑**		↑**		72	5
Distance moved		↓*		↓*		96	5
Thigmotaxis	↓**	↓**	↓**	↓**	↓**	96	5
Turn angle		↑**		↑**		96	5
Meander	↑*	↑**		↑*		96	5
Angular velocity		↑**		↑**		96	5
Clockwise rotations		↑**	↑*	↑**		96	5
Counter-Clockwise rotations		↑**		↑**		96	5
Velocity	↓*					96	5
Bursting frequency			↑*		↑**	96	5
Freezing frequency		↑**		↑**		96	5

* p < 0.05,

** p < 0.01 (Dunnett’s test)

**Table 3.** Chlorpyrifos behavioral results. Arrows indicate direction of change in comparison to vehicle control (↑increased or ↓decreased).

Parameter	Dark1	Light1	Dark 2	Light2	Dark 3	Exposure Time (h)	Concentration (μg l^−1^)
Thigmotaxis		↓**				48	0.05
Thigmotaxis			↑*	↑*		72	0.05
Thigmotaxis			↑*	↑*		96	0.05
Thigmotaxis	↑*					48	0.5
Clockwise rotations					↓*	48	5
Cruising frequency					↓*	48	5
Thigmotaxis	↑*					72	5
Meander	↑*					72	5
Cruising frequency	↓*		↓*			72	5
Bursting duration			↑**		↑*	72	5
Freezing frequency	↓*	↑*	↓*			72	5
Meander			↑**			96	5
Clockwise rotations				↑*		96	5
Freezing duration	↑*					96	5
Freezing frequency		↑*		↑**		96	5

* p < 0.05,

** p < 0.01 (Dunnett’s test)
