# Supplementary figures and images for: Exposure to permethrin or chlorpyrifos causes differential dose- and time-dependent behavioral effects at early larval stages of an endangered teleost species
Source: Endanger Species Res. Author manuscript; Available in PMC 2021 Aug 4. (PMC8336651; doi:10.3354/esr01091)

### A. Permethrin

Mean total distance moved (mm)

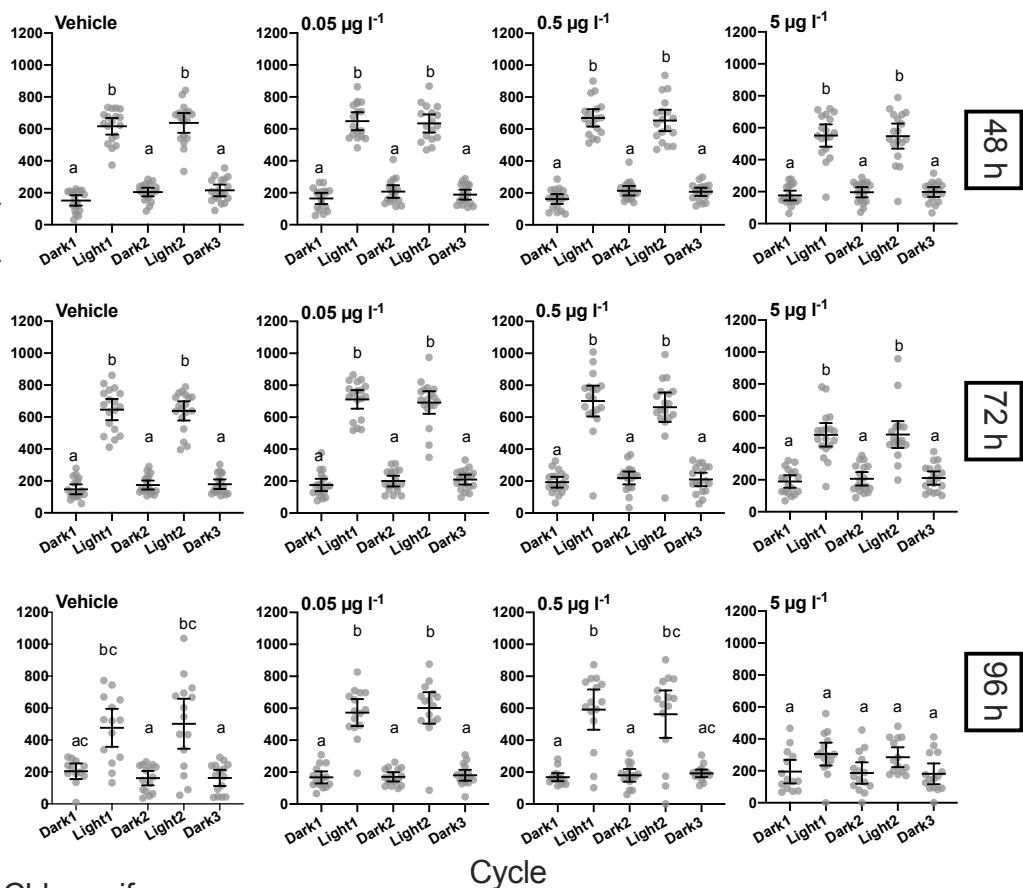

### B. Chlorpyrifos

Mean total distance moved (mm)

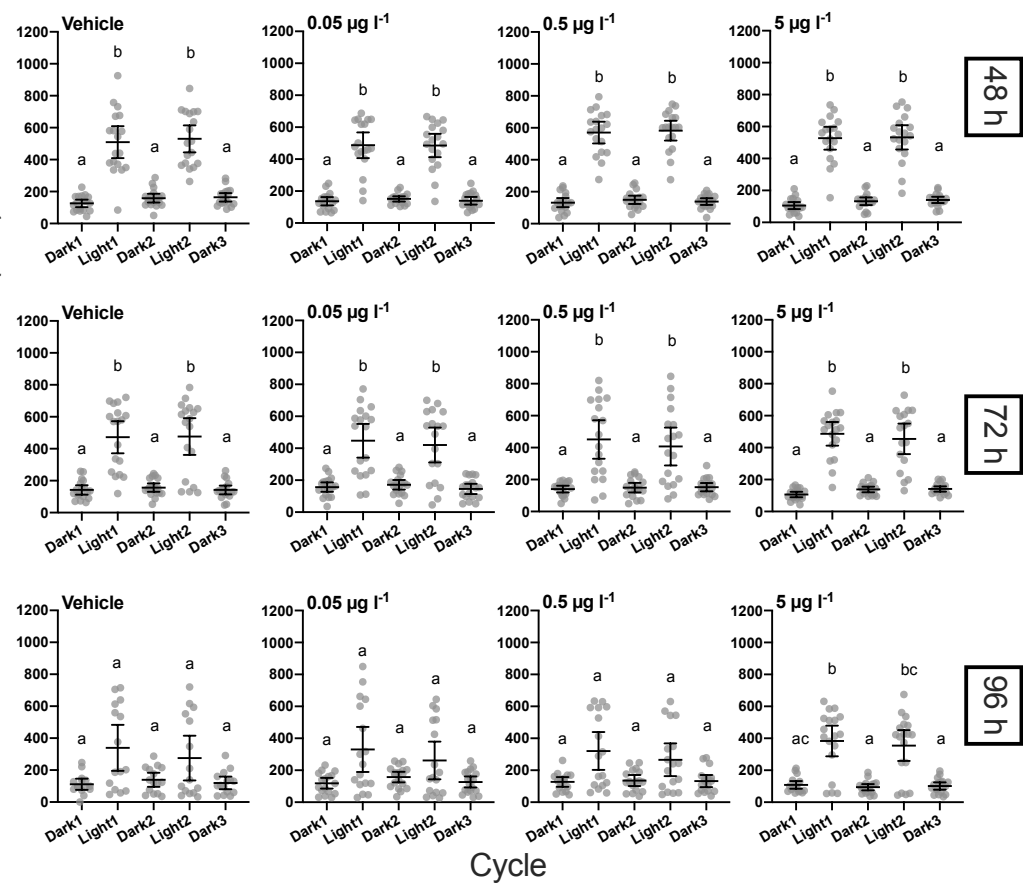

Supplement: Supplemental Figure 1 [file NIHMS1680042-supplement-Supplemental_Figure_1.pdf]

## A. Permethrin

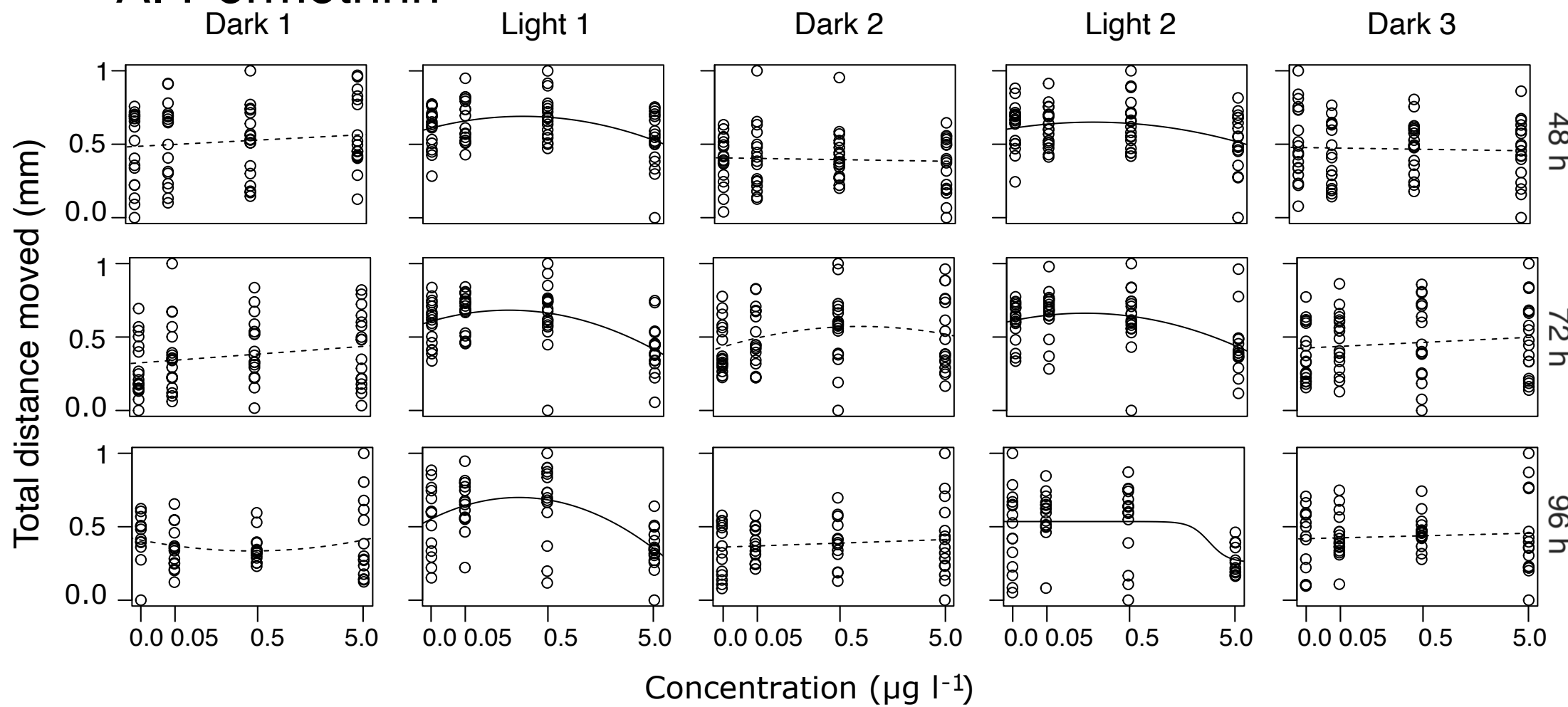

## B. Chlorpyrifos

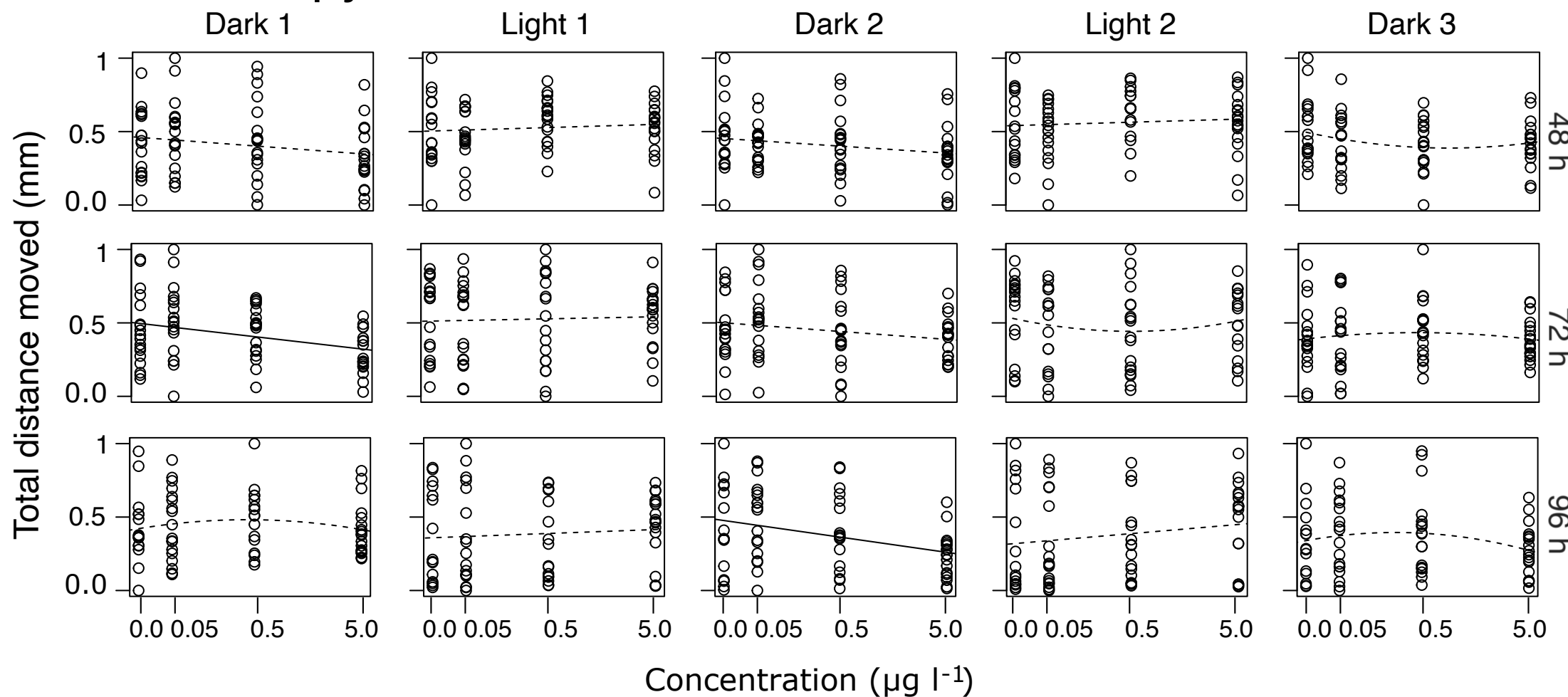

Supplement: Supplemental Figure 2 [file NIHMS1680042-supplement-Supplemental_Figure_2.pdf]
