# Supplementary material for: Exposure to permethrin or chlorpyrifos causes differential dose- and time-dependent behavioral effects at early larval stages of an endangered teleost species
Source: Endanger Species Res. Author manuscript; Available in PMC 2021 Aug 4. (PMC8336651; doi:10.3354/esr01091)
Supplement: Supplementary Material [file NIHMS1680042-supplement-Supplementary_Material.docx]

Exposure to permethrin or chlorpyrifos causes differential dose- and time-dependent behavioral effects at early larval stages of an endangered teleost species.

**SUPPLEMENTAL MATERIALS**

**Supplementary Methods**

Permethrin (100 µg/mL in acetonitrile, 39.8% cis-, 60.2% trans-) and chlorpyrifos (100 µg/mL in acetonitrile) were obtained from Sigma Aldrich (St. Louis, MO, USA). The recovery surrogate decachlorobiphenyl (DCBP; 200 µg/mL in nonane) was purchased from Supelco (Bellefonte, PA, USA). Stable isotope labeled internal standard D_6_-permethrin was donated by Kalexsyn, Inc. (Kalamazoo, MI, USA) and D_10_-chlorpyrifos, and ^13^C_12_-DCBP were obtained from Cambridge Isotope Laboratories (Tewksbury, MA, USA). HyperSep (3.0 mL/500 mg bonded-phase silica C_18_) solid phase extraction (SPE) cartridges, sodium sulfate (ACS grade), glacial acetic acid, and pesticide-grade solvents (hexane, methanol, and acetone) were obtained from Fisher Scientific (Pittsburgh, PA, USA). Nitrogen (99.998%) and helium (99.999%) were supplied by Airgas Inc. (Marion, IL, USA).

Water was spiked with the target analyte using acetone as the carrier at three concentration levels (low, medium, and high) and solvent control water was prepared as a blank. The exposure water concentration was measured by collecting three replicate 500 mL aliquots of water from 1000 mL beakers from each treatment. DCBP surrogate (40 ng) was added to each sample to estimate recovery, and methanol (5 mL) was added to each sample to improve extraction efficiency. Water was loaded onto a Hypersep SPE cartridge (10 mL/min) that previously had been conditioned with 3 mL of 1:1 hexane:acetone solution (volume:volume, v/v), methanol (3 mL) and deionized water (6 mL). After drying the cartridge for 20 min, the cartridge was eluted three times using 2.5 mL of a 1:1 hexane:acetone solution (v/v) and combined with triplicate hexane washes (1 mL) of the beakers. The eluate was evaporated under a gentle stream of nitrogen to 2 mL and the top organic layer was removed from the bottom aqueous later with three rinses with hexane (1 mL). The extract was passed through a sodium sulfate column (3 g previously dried 4 h at 400 ^o^C) to remove residual water. The extract was evaporated to 1 mL and acidified using glacial acetic acid (0.1% by volume, You and Lydy 2007). Prior to analysis, 20 ng of each internal standard was added.

Analysis was performed on an Agilent 6850A GC with a 5975C mass selective detector (MSD; Agilent Technologies; Santa Clara, California, USA) using methane negative chemical ionization (NCI) mode. Analytes and surrogate were separated using a HP-5 ms (30 m x 0.25 mm, with a 0.25 μm film thickness, Agilent) column. Helium was used as the carrier gas at a flow rate of 1.0 mL/min. A 2 μL sample was injected in pulsed splitless mode (2.1 x 10^5^ Pa for 0.75 min). The GC was programmed as follows: the oven was initially set at 50 °C, then ramped to 200 °C at 20° C/min, then heated to 295 °C at 10 °C/min and held for 10 min. The temperatures of the inlet, ion source, and quadrupole were set at 260 °C, 150 °C, and 150 °C, respectively.

Compounds were detected in selected ion mode, and internal standard calibration with multi-point standards (0, 1, 2, 5, 10, 50, 100, 250, 500 ng/mL) was used. Extracts were quantitatively diluted with hexane into the calibration range as needed. The identification of compounds was based on retention time (<0.1%), and confirmed using the ratio of the target and qualifier ions (<20%, Table S1) in comparison to standards. Permethrin was quantified as the sum of cis- and trans- isomers. The regression coefficient (r^2^) of the calibration curve was ≥ 0.998 for all analyses. The reporting limit for all analytes was set to 0.002 µg/L in water based on the lowest calibration standard. A matrix spike (MS) and matrix spike duplicate (MSD) were prepared and analyzed in each batch of extractions by spiking control water with 40 ng of permethrin and chlorpyrifos to verify the efficiency of the extraction process as percent recovery (acceptable recovery ranged from 70 to 130% with < 25% difference between the MS and MSD). A mid-range standard was analyzed once every eight samples to validate instrument performance (acceptable validation was < 25% difference). Extraction recovery for each sample was considered acceptable when percent recovery of DCBP relative to the spike check was between 70 and 130%.

Reference:

You, J and M.J. Lydy. 2007. A solution for isomerization of pyrethroid insecticides in gas chromatography. Journal of Chromatography A. 1166 (1-2), 181-190.

**Supplementary Tables**

**Table S1**. Optimized gas chromatography-mass spectrometry method parameters (methane negative chemical ionization mode). Permethrin was quantified as the sum of cis- and trans- isomers.

| Compound | MW  (g/mol) | *t*_R_  (min) | Target ion  (m/z) | Qualifier ion  (m/z) |
| --- | --- | --- | --- | --- |
| Chlorpyrifos | 350.6 | 10.98 | 313 | 315 |
| D_10_-chlorpyrifos (IS) | 360.6 | 10.92 | 323 | 325 |
| Permethrin | 391.3 | 16.28 cis/16.39 trans | 207 | 209 |
| D_6_-permethrin (IS) | 397.3 | 16.31 cis/16.42 trans | 213 | 215 |
| DCBP surrogate | 498.7 | 17.80 | 498 | 500 |
| ^13^C_12_-DCBP (IS) | 512.7 | 17.879 | 510 | 512 |

MW = molecular weight; *t*_R_ = retention time; DCBP = decachlorobiphenyl, IS = internal standard.

**Table S2.** Water chemistry results (mean and standard deviation of three samples).

| **Permethrin** | |
| --- | --- |
| **Nominal (µg l^-1^)** | **Measured (µg l^-1^)** |
| 0.05 | 0.052 ± 0.010 |
| 0.5 | 0.447 ± 0.048 |
| 5 | 3.860 ± 0.665 |
| **Chlorpyrifos** | |
| **Nominal (µg l^-1^)** | **Measured (µg l^-1^)** |
| 0.05 | 0.058 ± 0.024 |
| 0.5 | 0.589 ± 0.095 |
| 5 | 5.725 ± 0.655 |

**Supplementary Figures**

**Figure S1. Total distance moved in LD cycle assay during exposure to (A) permethrin or (B) chlorpyrifos.** Mean total distance moved over each cycle, of Delta smelt larvae at 48, 72, or 96 h of exposure (which correspond to 10, 11, or 12 dpf). Individual points represent larvae (n=15-18), and bars represent mean and 95% confidence interval. Compact letter display represents groups (mean total distance moved over entire cycle) significantly different from each other in Dunn’s multiple comparisons test, p<0.05.

**
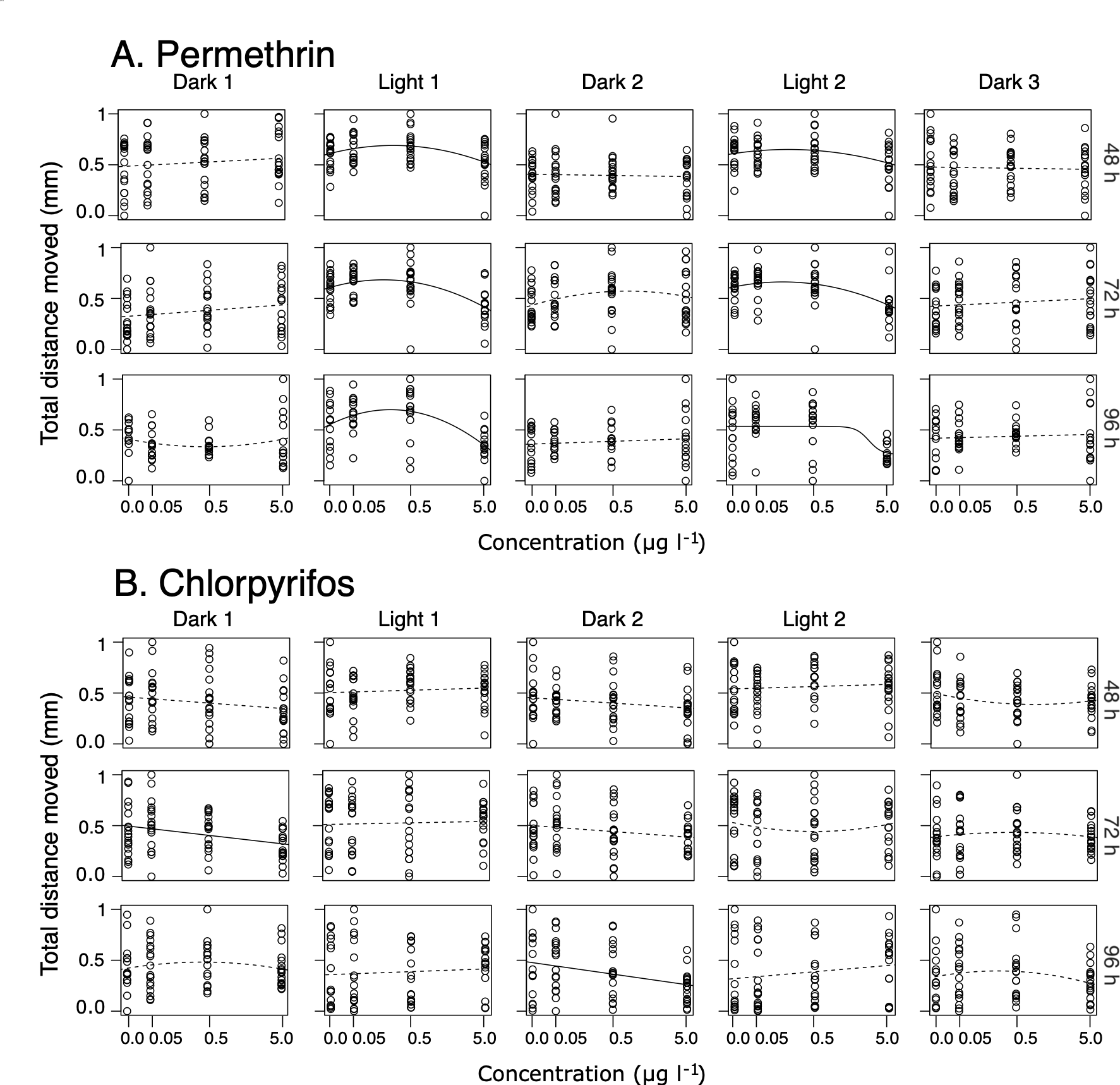
**

**Figure S2.** Mean total distance moved was rescaled between 0 and 1 within each cycle to facilitate comparison between pesticide concentration [(**A**) Permethrin, (**B)** Chlorpyrifos] within each cycle. Each circle represents the rescaled mean total distance moved of one larva (n=18). Data are presented on a log10 X+ 0.05 axis. For each panel, the data was assessed being fit to five curves (linear, quadratic, sigmoidal, unimodal1, and unimodal2) using a maximum likelihood approach. The best fitting curve is shown in each panel. Curves shown as a solid line are significantly better fits than a null intercept-only model (p<0.05), curves shown as a dashed line are the best-fit of the five-curve option (lowest p-value), but not significantly better than the null model.
